# Morphology of the human inner ear and vestibulocochlear nerve assessed using 7 T MRI

**DOI:** 10.1007/s10334-024-01213-3

**Published:** 2024-11-13

**Authors:** Kingkarn Aphiwatthanasumet, Ketan Jethwa, Paul Glover, Gerard O’Donoghue, Dorothee Auer, Penny Gowland

**Affiliations:** 1 Sir Peter Mansfield Imaging Centre, School of Physics and Astronomy, University of Nottingham, Nottingham, UK; 2 Department of Radiology, Nottingham University Hospitals NHS Foundation Trust, Nottingham, UK; 3 Department of Otolaryngology, Head and Neck Surgery, University of Nottingham, Nottingham, UK; 4 Sir Peter Mansfield Imaging Centre, School of Medicine, The University of Nottingham, Nottingham, UK; 5 Department of Radiological Technology, Faculty of Allied Health Sciences, Naresuan University, Phitsanulok, Thailand

**Keywords:** Inner ear, Adult cochlea, Cochlear structure, Vestibulocochlear nerve, Dielectric pads

## Abstract

**Objective:**

To optimize high-resolution 7 T MRI of the cochlea and measure normal cochlea and the cochlear nerve morphometry in vivo.

**Materials and methods:**

Eight volunteers with normal hearing were scanned at 7 T using an optimized protocol. Two neuroradiologists independently scored image quality. The basal turn lumen diameter (BTLD), height, width, length and volume of the cochlear, long (LD) and short (SD) diameter the calculated cross-sectional area (CSA) of the cochlear nerve were measured. Intra and inter-observer reliability was assessed using intraclass correlation (ICC).

**Results:**

3D T2W DRIVE combined with dielectric pads, allowed acquisition of high-resolution images showing detailed structures, such as the crista ampullaris in the semicircular canals. The overall grading scores from neuroradiologists were excellent. In the left ear, averaging over all subjects gave BTLD of 2.6 ± 0.05 mm, height of 4.9 ± 0.1 mm, width of 4.4 ± 0.2 mm, length of 36.5 ± 0.4 mm, volume of 0.16 ± 0.02 ml, LD of 1.31 ± 0.1 mm, SD of 1.06 ± 0.1 mm, and CSA of 1.1 ± 0.1 mm^2^. The right ear gave BTLD of 2.6 ± 0.04 mm, height of 4.9 ± 0.1 mm, width of 4.4 ± 0.3 mm, length of 35.5 ± 0.4 mm, volume of 0.16 ± 0.02 ml, LD of 1.29 ± 0.1 mm, SD of 1.07 ± 0.1 mm, and CSA of 1.10 ± 0.2 mm^2^. No statistically significant difference was found between the sides of the head (*p*-value > 0.05). The intra-observer reliability was high (0.77–0.94), while the inter-observer reliability varied from moderate to high (0.55–0.81).

**Conclusion:**

7 T MRI can provide excellent visualization of the internal structure of the cochlear and of the vestibulocochlear nerve in vivo.

**Supplementary Information:**

The online version contains supplementary material available at 10.1007/s10334-024-01213-3.

## Introduction

Magnetic resonance imaging (MRI) can assess the membranous labyrinth within the bony labyrinth and its anatomical relations. Cochlear anomalies, ossification, and deficiencies in the cochlear nerve are causes of hearing loss and detailed imaging to measure cochlear size has proven valuable for planning cochlear implant surgery [1]. Atrophy or change of the cochlear nerve is a primary concern for individuals with auditory neuropathy [2–4]. Moreover, identification of patients with disorders of the cochlear nerve such as aplasia or hypoplasia is crucial in their assessment for cochlear implantation. Thus, the detailed visualization of cochlear microstructure and the cochlear nerve is central to surgical planning in patients with profound hearing loss as findings may impact on their candidacy and may also influence device selection.

Ultra-high field MRI offers greater sensitivity compared to lower field MRI [5–7] and the resulting enhancements in visualization of detailed anatomy may offer potential improvements in visual diagnosis and treatment decision-making. However, field inhomogeneities remain a major challenge at ultrahigh field. We anticipate that MR imaging protocols optimized for resolution, contrast and scan time will provide an opportunity to enhance our understanding of anatomical variations in the adult cochlea by measuring size and length relative to the surrounding cranial nerves.

The aims of this study were: (i) to optimize an MR protocol for high-resolution inner ear imaging using 7-Tesla MRI, and (ii) to use this protocol to measure the normal dimensions of the human cochlea and vestibulocochlear nerve.

## Methods

First T2-weighted imaging of the inner ear imaging was optimized, including the use of dielectric pads to improve B1 inhomogeneity and Maximum Intensity Projections (MIPs) to enhance visualization. Next two neuroradiologists independently scored image quality. Finally, cochlea and vestibulocochlear nerve morphology were measured, and anatomical variability assessed in subjects with normal hearing.

### Subjects

This study was approved by the University of Nottingham, Faculty of Medicine and Health Sciences Research Ethics Committee (H14082014/22). Eight healthy subjects aged between 25 and 55 years with normal hearing and no ear diseases underwent screening with MR safety questionnaires and gave written informed consent before every scan.

### Image acquisition

MR images acquired in three planes (i.e., axial, oblique coronal and perpendicular to cochlear structure) at 7 T using a Philips MR scanner (Achieva) equipped with two-channel and eight-channel transmit system head coils (Nova Medical Inc., USA), although we primarily used the two-channel system in quadrature mode for this study. In three subjects, we performed a preliminary comparison of the Fast Spin Echo (FSE), balanced Steady State Free Precession (bSSFP) and 3D-DRIVen Equilibrium (3D-DRIVE) sequences at 7 T. The DRIVE sequence is a FSE sequence which includes a 90⁰ reset pulse after the FSE readout to speed up longitudinal recovery between shots [8, 9]. The range of sequence parameters considered, and the final optimum selected are shown in Table 1. To improve B1, we investigated the use of dielectric pads (18 × 18 cm, containing a suspension of calcium titanate with a 2.8:1 weight ratio in deuterated water) placed over both ears [10–13].

**Table 1:** MR sequences for high-resolution cochlear imaging; the final optimized values at 7 T are indicated in bold font

Field strength	7 T		
MR sequence	FSE	bSSFP	DRIVE
Scan mode	3D	3D	3D
Voxel size (mm)	0.4 × 0.4x0.8	0.4 × 0.4x0.8	0.2 × 0.2x0.4–**0.26 × 0.26x0.26**
Matrix size	412 × 412	384 × 356	800 × 800–**896 × 896**
Slices	30	100	45–**76**
FOV (mm) (AP, FH, RL)	139 × 30x165	140 × 40x164	166 × 18x160–**224 × 20x164**
SENSE (RL, FH)	2, 0	2, 0	2, 0
Echo train length (ETL)	50	-	50
Flip angle (°)	90	40	**100**
TR (ms)	2500	11	4500–**3300**
TE (ms)	395	5.6	220–**282**
Scan time (min)	6:42	3:45	6:53–**14:47**

### Image analysis

Although this study did not measure image contrast and SNR, image quality was assessed and scored by two neuroradiologists using the following scale: 0-not visible; 1-partially visible; and 2-completely visible.

Maximum intensity projections were used to visualize small cochlear structures and determine the normal variations in shape and size among eight subjects. The cranial nerves (facial nerve (CN VII) and vestibulocochlear nerve (CN VIII)), were evaluated on left and right sides of the head for the degree of variation among and between subjects. Data were analysed using MATLAB (version 2021a) and FSL software packages.

**Fig. 1:**
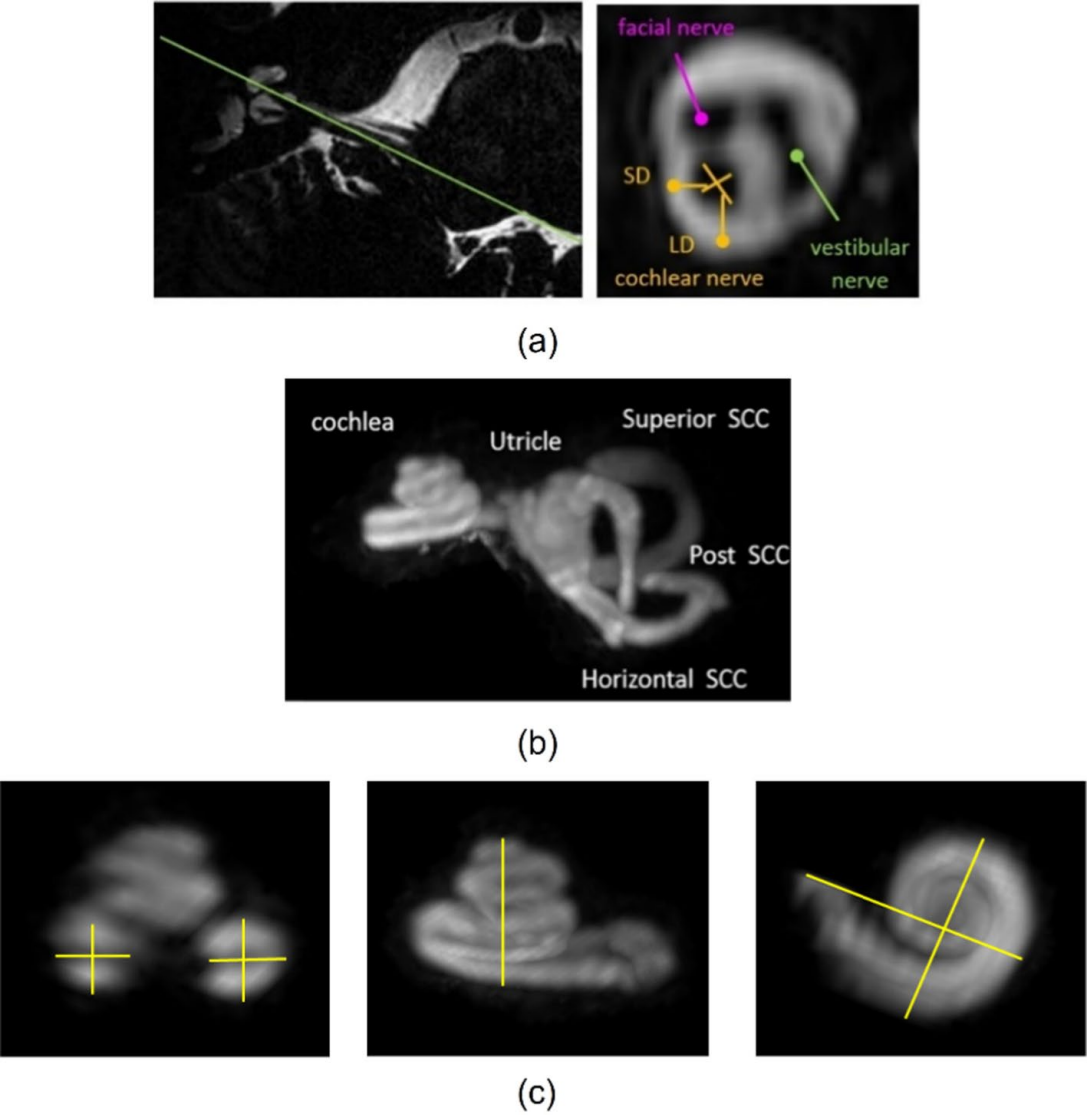
**a** Indicating measurements of the long diameter (LD), short diameter (SD), and cross-sectional area (CSA) of the cranial nerves. **b** MIP images of inner ear (from subject 1) where the semi-circular canals connect to the cochlea at 7 T. **c** indication of measurement of cochlear dimensions including BTLD, height, width, and length using MIP images

The measurements of cochlear dimensions included the basal turn lumen diameter (BTLD), height, width, length, and volume of the cochlea. These were determined using the method described by Heutink et al. [14] and Pelliccia et al. [15]. Results from each measurement were presented as mean ± SD to assess the variability in cochlear size across both ears of the subjects. As shown in Fig. 1, the thickness of the cochlear nerve was measured by defining the long diameter (LD) short diameter (SD) and hence calculating cross-sectional area, $CSA=\pi \left(\frac{LD}{2}\right)\left(\frac{SD}{2}\right)$. For measurements of cochlear dimension, one observer measured the MR images twice to assess intra-observer reliability, while the measurement of the cochlear nerve was conducted by two observers. Intra-observer and inter-observer reliability were assessed using the intra-class correlation coefficient [16]. A p < 0.05 was considered statistically significant, and all reliability assessments were presented with a 95% confidence interval (CI).

## Results

### Optimising the imaging sequence

**Fig. 2:**
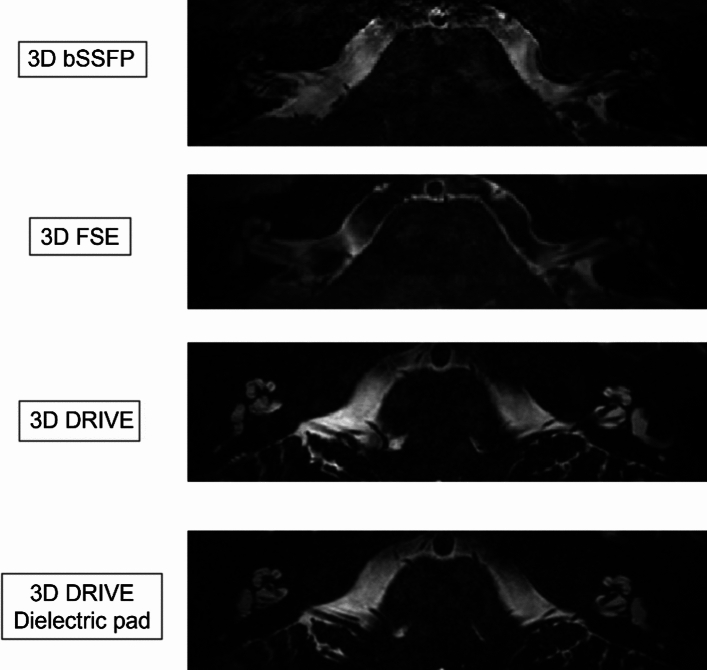
Comparison of MR sequences (3D bSSFP, 3D FSE, and 3D DRIVE) for cochlear imaging at 7 T and effect of dielectric pads on 3D DRIVE

**Fig. 3:**
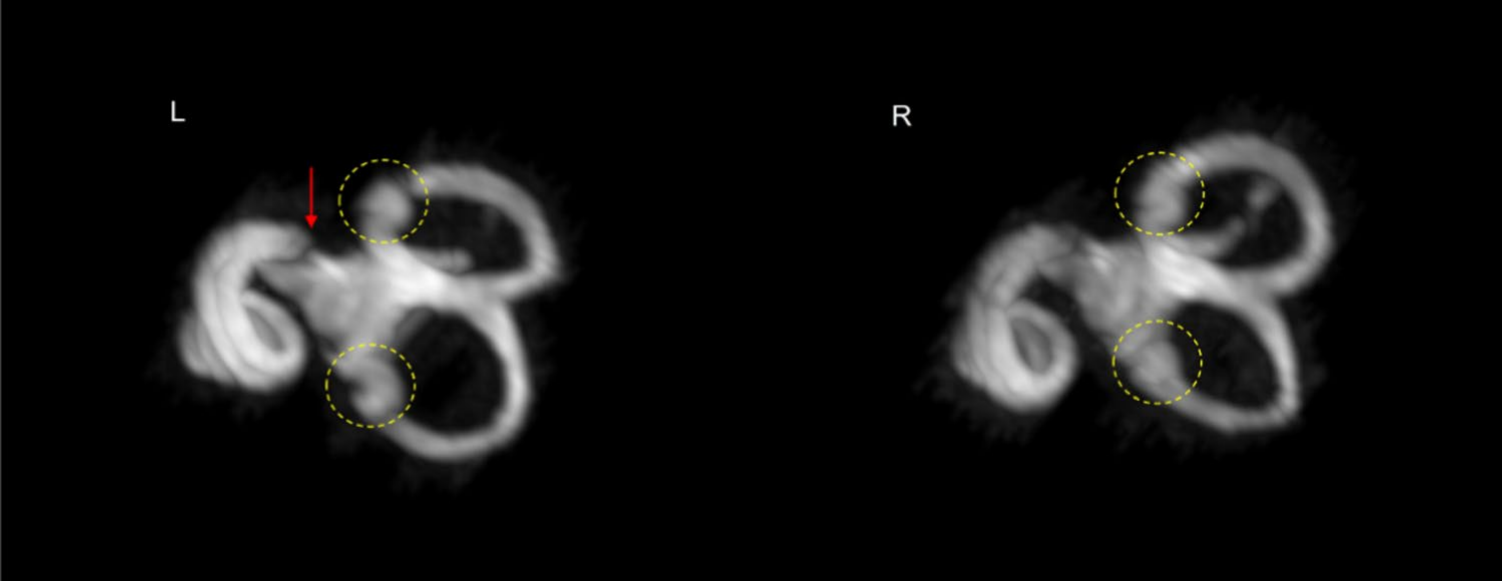
High-resolution MR images of the inner ear obtained using 3D T2W DRIVE and dielectric pads show improved visualization of the vestibular system. The crista ampullaris in the semicircular canals is indicated by yellow circle. The link between the saccule and vestibular duct is also visible on the left hand side (red arrow, see Supplementary material in Fig. 1 for a different projection in which this is clearer)

**Fig. 4:**
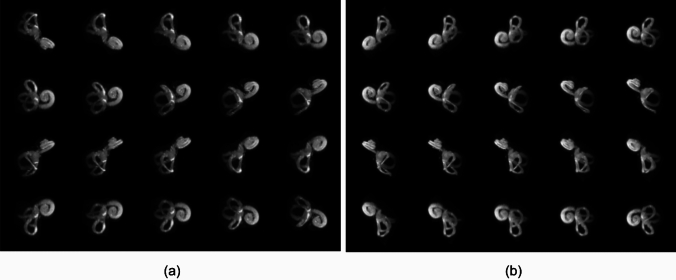
MIPs of images of the **a** right and **b** left inner ears of one subject (12,103) shown at different projection angles, where the semicircular canals connect to the cochlea acquired using 3D T2W DRIVE and dielectric pads at 7 T MRI

The semicircular canals, cochlear structures and the cranial nerves were most clearly depicted in 3D DRIVE images and image homogeneity was improved by using dielectric pads (Fig. 2, 3, 4).

### Imaging of the inner ear structures, including the semicircular canals

The 7 T MRI resulted in improved visualization of three fluid-filled canals of the cochlea with 3D DRIVE and dielectric pads. These results provided greater anatomy of the crista ampullaris in the ampullae of all three semicircular canals. The details of these structure are presented in Figs. 3, 4.

### Imaging of the CN VII and CN VIII

**Fig. 5:**
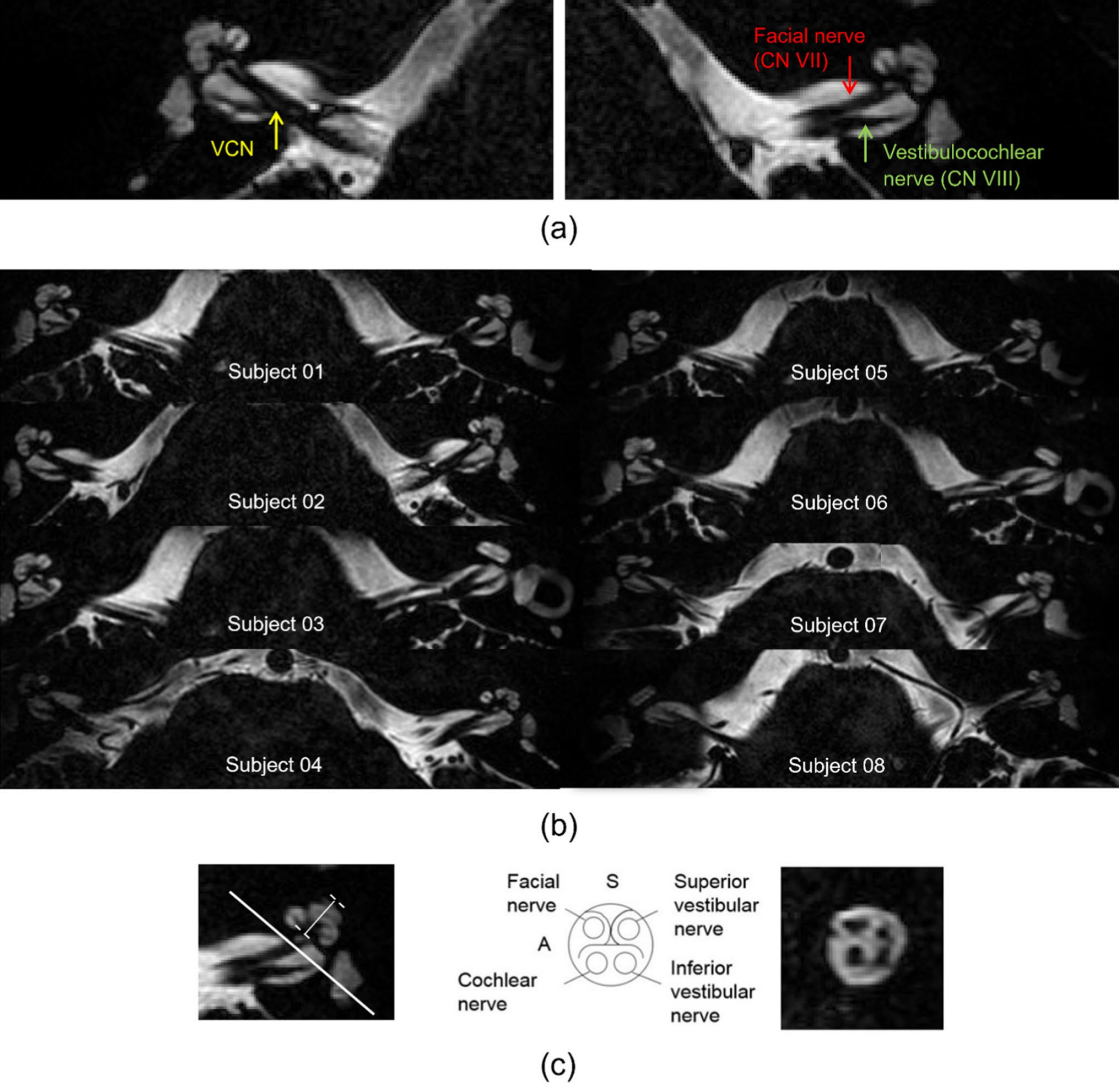
**a** MR images acquired with dielectric pads, show both sides of the cochlea and the branches of cranial nerves (CN VII, VII). The branching of the vestibulocochlear nerve (VCN) is indicated by the yellow arrow, CN VII by the red arrow, and CN VIII by the green arrow. **b** The normal anatomy of cranial nerves in the ear canal across eight subjects, which was clearly visible on axial slices at 7 T MRI. **c** The height measured in the IAC and the cranial nerves (CN VII, VII) on an axial image, running perpendicular to the internal auditory canal

Figure 5 shows the vestibulocochlear nerve (CN VIII), located posterior to the facial nerve (CN VII) and running laterally to the ear canal; the visibility of cranial nerves was excellent in the plane perpendicular to the internal auditory canal.

### Imaging of the cochlea

**Fig. 6:**
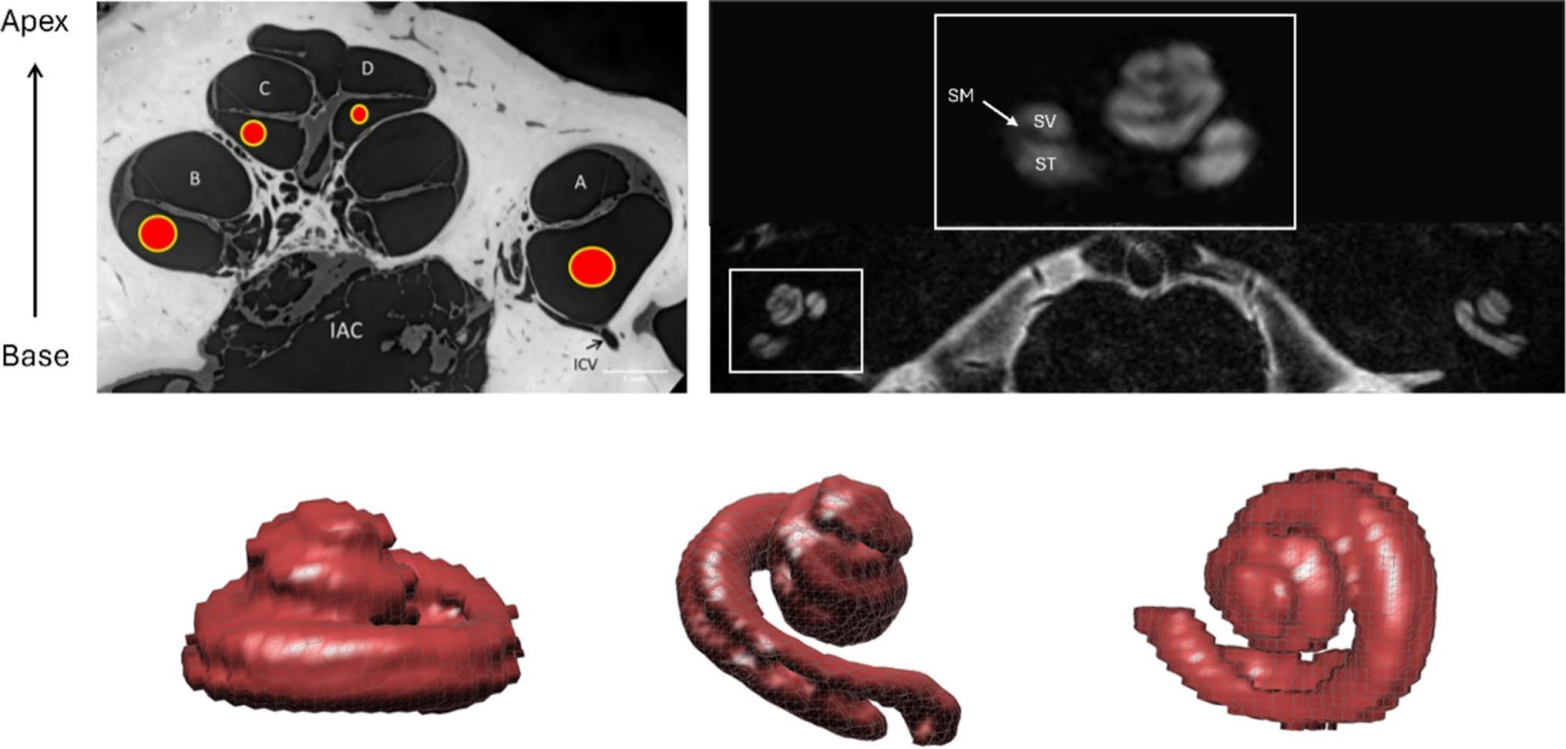
Synchrotron radiation phase-contrast imaging (SR-PCI) section from Giese et al. [17] and 7 T 3D T2W DRIVE image including constructed orthogonal 3D volume renderings of the cochlea; MOD: modiolus, *BM* basilar membrane, *RM* Reissner’s membrane, *ST* scala tympani, *SM* scala media, and *SV* scala vestibule

In general, the cross-sectional MR image of the cochlea was well-defined the cochlear canals (Fig. 6), and internal structures of the cochlea such as the modiolus, scala tympani (ST), scala media (SM), scala vestibule (SV), were all detected at 7 T in all subjects. The low signal band between SV and ST likely represents the SM. However, some structures, including Reissner’s membrane and the basilar membrane, were not visible at 7 T MRI.

### Clinical image quality assessment

**Fig. 7:**
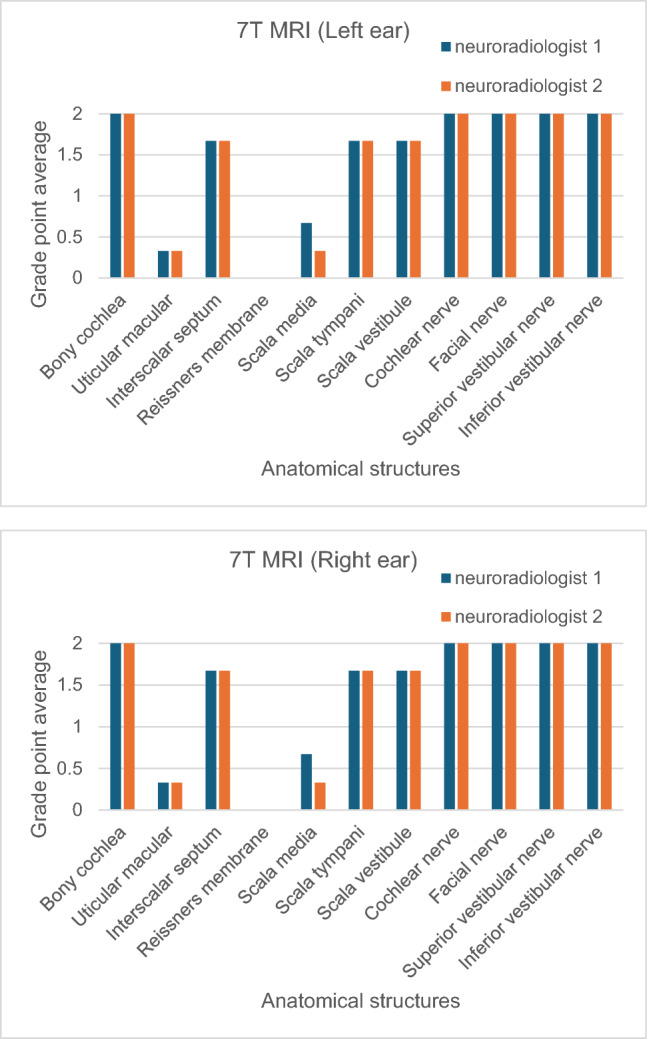
Image quality scored by two neuroradiologists at 7 T MRI

Figure 7 shows that the neuroradiologists considered that the bony cochlea (from the base to the apex) and nerves were completely visible (scores of 2). The utricular macular, interscalar septum, scala media, scala tympani, and scala vestibule were scored 1 or 2. The Reissner’s membrane could not be imaged (scores of 0). The study showed an excellent intra-observer reliability, with ICC values of 0.90 (95% CI = 0.64–0.98) for observer one and 0.88 (95% CI = 0.56–0.98) for observer two. The inter-observer reliability showed ICC values of 0.95 (95% CI = 0.87–0.99), indicating excellent reliability among observers (Table 2).

**Table 2:** Inter-observer and intra-observer reliability of the inner ear were assessed using the intra-class correlation coefficient (ICC) at 7 T MRI

The cochlea and nerves	Inter-observer ICC (95% CI)	Intra-observer ICC (95% CI)	
		Observer 1	Observer 2
Left ear	0.955 (0.871–0.991)	0.903 (0.637–0.982)	0.882 (0.559–0.978)
Right ear	0.955 (0.871–0.991)	0.903 (0.637–0.982)	0.882 (0.559–0.978)

### Measurements of key structures in the internal auditory canal and inner ear

The cochlea spirals 2.5 turns around its axis. The results of key measurements made in this study are shown in Table 3 and demonstrate high intra-observer reliability for both sides. The values of ICC ranged from 0.82 to 0.92, reached 0.92 (95% CI = 0.83–0.95) on the left, and 0.82 (95% CI = 0.78–0.93) on the right.

**Table 3:** Cochlear dimensions measured on both sides for eight subjects with normal hearing. A single observer performed the measurement to assess intra-observer reliability

Cochlear dimensions	Left side	Right side
Length of cochlear duct	36.5 ± 0.4 mm	35.5 ± 0.4 mm
Width of cochlear duct	4.4 ± 0.2 mm	4.5 ± 0.3 mm
Height of cochlear duct	4.9 ± 0.1 mm	4.9 ± 0.1 mm
Basal lumen diameter	2.6 ± 0.05 mm	2.6 ± 0.04 mm
Volume of cochlear duct	0.16 ± 0.02 ml	0.16 ± 0.02 ml
Intra-observer ICC (95% CI)	0.92 (0.83–0.95)	0.82 (0.78–0.93)

Table 4 presents measurements of the four nerves in the internal auditory canal (IAC) and shows that the ICC for intra-observer reliability tended to be higher than that for inter-observer reliability. There was no significant difference in any measurement between each side of the head (*p*-value > 0.05).

**Table 4:** The cochlear nerve diameter and variability measures between observers were assessed across the eight subjects with normal hearing, with 95% confidence interval

Observer	LD (mm)		SD (mm)		CSA (mm^2^)	
	Left	Right	Left	Right	Left	Right
Observer 1	1.32 ± 0.07	1.28 ± 0.11	1.08 ± 0.10	1.07 ± 0.13	1.12 ± 0.14	1.08 ± 0.18
Observer 2	1.31 ± 0.06	1.30 ± 0.09	1.05 ± 0.04	1.07 ± 0.09	1.08 ± 0.06	1.09 ± 0.10
Average	1.31 ± 0.1	1.29 ± 0.1	1.06 ± 0.1	1.07 ± 0.1	1.10 ± 0.1	1.10 ± 0.2
Inter-observer ICC	0.744 (0.541–0.943)	0.694 (0.421–0.943)	0.547 (0.341–0.898)	0.811 (0.638–0.957)	0.690 (0.419–0.930)	0.594 (0.407–0.909)
Intra-observer ICC (observer1)	0.843 (0.515–0.969)	0.836 (0.485–0.964)	0.905 (0.527–0.981)	0.938 (0.690–0.988)	0.873 (0.564–0.974)	0.834 (0.473–0.967)
Intra-observer ICC (observer2)	0.800 (0.609–0.950)	0.768 (0.541–0.947)	0.866 (0.663–0.973)	0.848 (0.591–0.981)	0.869 (0.649–0.930)	0.777 (0.681–0.954)

## Discussion

The heavily T2-weighted 3D DRIVE sequence provided apparently superior image quality compared to the 3D bSSFP and 3D FSE sequence at 7 T, providing information on detailed anatomical structures of the inner ear, including the three internal chambers of the cochlea, three semi-circular canals (SCC), and also the cranial nerves (cochlear nerve, superior and inferior divisions of the vestibular nerve (CN VIII) and facial nerve (CN VII)). We observed that, the facial nerve and superior vestibular nerve were located in the superior quadrants of the IAC, while the cochlear nerve and inferior vestibular nerve were situated in the inferior half of the IAC, consistent with previous studies [18, 19]. The cochlear nerve has previously been studied at 3 T for instance in health [20] and in case of suspected absence of the nerve [21], and although quantitative comparisons are difficult between, he papers, the spatial resolution achieved was higher at 7 T, and the image SNR appears higher. 7 T MRI has the potential to provide further information for clinical practice, and previous work has indicated that improved diagnosis can be achieved with 7 T MRI [7, 22, 23].

Although van der Jagt et al. [7] were the first to present in vivo evidence of the scala media (or cochlear duct) using state-of-the-art 7 T MRI to demonstrate high-resolution inner ear anatomy, our study focused on optimizing an MR protocol specifically to measure the normal dimensions of the human cochlea and vestibulocochlear nerve. This may prove helpful in the future evaluation of vestibular disorders. We also successfully imaged the crista ampullaris within the ampullae of all three semicircular canals (Fig. 3). This contains vestibular receptors, a group of hair cells that respond to head movement and rotation [24–26]. Such imaging may help investigate vertigo and in particular the vertigo experienced by strong magnetic fields (as it will be possible to relate the direction of the field to the positions of the vestibular components).

The use of dielectric pads has previously been shown to improve the transmit field performance over the inner ear regions in both single transmit mode (1Tx/32Rx RF coil) and parallel-transmit mode (8Tx/32Rx dipole coil array) [27]. However, in this study, we achieved better of image quality, including higher spatial resolution and shorter scan time possibly partly because of the difference in size of the pads used, as well as because of differences in imaging sequences. A previous study has proposed using dielectric pads tailored to optimising the B1 in the inner ear region [28] which may produce improved results, although this study has shown that good results can be obtained using generic pads.

Within the IAC, there are three separate nerves: facial nerve, cochlear nerve, and vestibular nerve have been assessed. Measurements of these cranial nerves using 7 T MRI showed that the size of the facial nerve and the vestibulocochlear nerve similar, in line with those observed in earlier study. A case study by Kim et al., found that the cochlear branch was identified as the largest segment of the VCN, while the inferior vestibular branch was the smallest segment on MR images (88% of the 58 cases). Among the 23 healthy participants, the relative size of these nerves showed symmetry in 70% of cases and varied among individuals depending on the location [29]. Furthermore, in a case report of cochlear nerve aplasia or hypoplasia, Furuta et al. [4] reported that the cochlear nerve was smaller than the facial nerve, attributed to infection of the inner ear and associated with a decline in hearing ability. Overall, this study strengthens the idea that MRI holds great potential for detecting the VCN abnormality which helps in selecting candidates for cochlear implant surgery.

This study characterized the geometry of the cochlea in normal subjects. Our results align with similar measurements of dimensions previously reported in high-resolution computed tomography (CT) images in vivo [15, 30, 31]. This information is crucial to the imaging of the morphological aspects of inner ear disease. For future cochlear implant candidates, high-resolution MRI combined with 3D printing technology could be used to create simulations for surgical planning and patient-specific preparation. We also recommend the exploration of additional imaging planes, such as oblique-coronal or oblique-sagittal views, which could be beneficial for detecting pathology within the internal auditory canal. Furthermore, if MRI can detect the onset of ossification, it may eliminate the need for CT scans in young children, making MRI the imaging method of choice with associated reduction in radiation exposure.

## Conclusions

The modified 3D T2W DRIVE sequence, coupled with two-channel RF shimming and dielectric pads, facilitates the acquisition of high-resolution images of the inner ear, the internal cochlear chambers, the three semicircular canals, and cranial nerves. This is achieved using an isotropic voxel size of 0.26 mm, all completed in a scan time of less than 15 min.

## Supplementary Information

Below is the link to the electronic supplementary material.

Supplementary file1 (DOCX 26 kb)

## Data Availability

The data that support the findings of this study are not openly available due to reasons of sensitivity and are available from the corresponding author upon reasonable request. Data are located in controlled access data storage at Sir Peter Mansfield Imaging Centre.
