# Supplementary material for: Morphology of the human inner ear and vestibulocochlear nerve assessed using 7 T MRI
Source: MAGMA. 2024 Nov 13;38(1):121–30. doi: 10.1007/s10334-024-01213-3 (PMC11790716; doi:10.1007/s10334-024-01213-3)

**Supplementary material**

**Fig 1.** An example of the image from another orientation shows the apparent discontinuity where the cochlear duct joins the saccule.


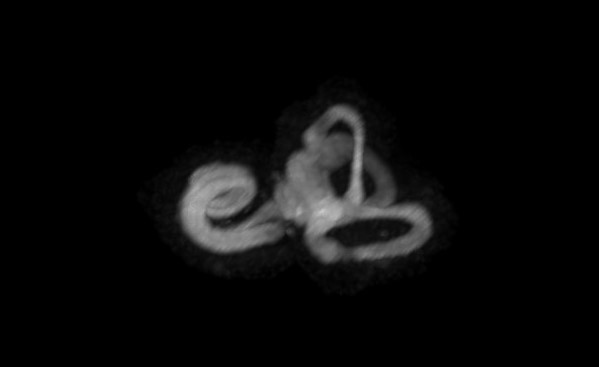

Supplement: Supplementary file 1 — Supplementary file1 (DOCX 26 kb) [file 10334_2024_1213_MOESM1_ESM.docx]
